# Research approvals iceberg: helping it melt away

**DOI:** 10.1186/s12910-019-0434-2

**Published:** 2019-12-24

**Authors:** Simon E. Kolstoe, David Carpenter

**Affiliations:** 1 School of Health Sciences and Social Work, University of Portsmouth, Portsmouth, PO1 2UP UK; 2 Independent consultant and trainer in research ethics, Portsmouth, UK

**Keywords:** Research ethics, Governance, Trials, Ethics committee, REC

## Abstract

**Background:**

In their paper “Research approvals iceberg: how a ‘low-key’ study in England needed 89 professionals to approve it and how we can do better” Petrova and Barclay highlight concerns with the health research regulatory environment in the UK.

**Discussion:**

As long-standing chairs of NHS research ethics committees, researchers, and also academics in research ethics, we are also often frustrated with the regulatory process in the UK. However, we think that Petrova and Barclay’s analysis is misleading because it conflates research ethics with governance and funding processes, thus failing to adequately distinguish between the national coordinating function of the Health Research Authority, local research governance processes, and interactions with research sponsors and/or the Clinical Research Network.

Holding regulatory authorities and systems to account is an important and valid task. As research is an inherently complex and difficult process, more must and can always be done to support academics and clinicians to successfully navigate the landscape. Layers of complex bureaucracy do not help anyone. Yet critical historical failings and criminal activities have created the need for robust integrity, ethics and governance processes. The challenge is to find the balance between a system able to effectively prevent the failings of the past, and yet at the same time not turn the research process into an impenetrable quagmire that inhibits the production of important knowledge. The first aim of regulatory processes is therefore “*to protect and promote the interests of patients and the public”* [1], but the second should be to promote and support good research. Critically reviewing and quantifying the research regulatory processes so as to hold systems and administrators to account is an important task. In their paper “Research approvals iceberg: how a ‘low-key’ study in England needed 89 professionals to approve it and how we can do better” [2] Petrova and Barclay provide a brief review of the literature on “RECs (Research Ethics Committees), IRBs (Institutional Review Boards) and ethics approvals for health research” before attempting to list the individuals, exchanges and emails required to get a single project underway in the UK’s National Health Service (NHS) setting. As described the results are shocking, but the paper contains a key misunderstanding by conflating different systems and processes.

## Distinguishing between research ethics and research governance

In 2017 the UK House of Commons Science and Technology Select Committee launched an inquiry into research integrity [3]. This was partly in response to an influential 2014 report by the Nuffield Council on Bioethics [4] examining the culture of research in the UK in an attempt to address “*fears that the ‘publish or perish’ culture leads to poor or questionable research practices*” [5]. Over 80 written submissions were made to the select committee that were subsequently made available on the committee website [6]. One of us (SEK) used these submissions as raw data for training students in the conduct and use of basic content analysis. Although this analysis was conducted as a training exercise not intended for publication, it was found anecdotally that there was significant confusion in the submissions between the terms “governance”, “integrity” and “ethics”. This was notable because submissions to the inquiry were made by research councils, universities, charities, learned organisations, academics and groups with a strong interest in the scientific method and process; in other-words expert organisations that were familiar with the practice, social and political aspects of Science as practised in the UK. This is concerning because it leads to the conflation of functions and roles, and significant confusion as to who is responsible, and ultimately accountable, for different parts of the system. The paper by Petrova and Barclay reflects this confusion, and provides a helpful opportunity to illustrate, in particular, the differences between research governance and research ethics.

To their credit Petrova and Barclay do refer to both ethics and governance in their abstract and introduction, followed by stating:

*A further clarification of scope and terminology may be helpful. Most of the background literature addresses the ethics approvals of studies. However, study approvals are a broader enterprise, variably termed (at least in the UK) “research ethics and governance approvals”, “ethics and R&D (research and development) approvals”, “study assurances”,* etc. *Additional sign-offs are needed by R&D departments of participating organisations or university research offices (if the ethics approval has been granted by a committee unattached to a university) around compliance with regulations or the capacity of an organisation to host a project. Researchers involved in human subjects research often use “ethics approvals” as a synecdoche for this broader class of research ethics and governance approvals, not least because the latter are typically contingent on the former and because the bulk of the documentation is first prepared for the ethics review…*

The authors’ recognition of this distinction between ethics and governance is extremely important, so it is disappointing that they finish this paragraph by stating that they will conflate the two as:

*… Our paper concerns this broader class of approvals.*

This is a significant mistake as it fails to recognise the distinct roles of governance and ethics, and the fact that each has a slightly different philosophical and practical contribution to the conduct of “good science”. Indeed it is one of the strengths of the UK system that ethics review and governance processes are separated, unlike the Institutional Review Board (IRB) system in the United States whose main weakness (in our opinion) is also conflating these two areas. We therefore agree with them that “ethical dilemmas are amongst the most engaging and enlivening topics of conversation” because this is precisely our experience of chairing research ethics committees. However, we disagree that the role of research ethics committees in particular is “hardly ever about ethical deliberation” or “caring about ethical conduct only insofar as litigation and reputational damage can be avoided”. This may (or may not) be true of the regulatory process overall, but certainly does not accurately describe the attitude or experience of research ethics committees.

### Research ethics

Within the current NHS system the task of the research ethics committee is the review of a specific protocol by up to 16 individuals consisting of both lay and expert members. We have previously described both the content and weaknesses of such reviews (and suggested areas for further research into how RECs make decisions) [7], but essentially these committees have the role of assessing whether the project, if conducted as described in the protocol, Integrated Research Application System (IRAS) form and related documentation [8], is ethical; firstly for the participants and secondly within the broader context of health and social care research. As this judgement necessarily appeals to moral norms, it necessarily must be conducted by a committee of individuals constituted with (at least approximate) knowledge of current normative ethical values. This is a complex task with the possibility of inconsistency, and it is notable how well the UK Health Research Authority (starting under its previous guise as the National Research Ethics Service (NRES)) has been able to organise and guide such reviews thanks to extensive training of committee members, guidance such as the Governance Arrangement for Research Ethics Committees (GAfREC) [9] and Standard Operating Procedures (SOPs) [10]. Additionally it should also be noted that RECs are made up of volunteers often reviewing studies in their spare time and not always supported by their employers. HRA coordinated RECs now review projects within strict timeframes: 60 days for full review of complex projects or 21 days for simpler ones. These timeframes are monitored by a virtual clock to ensure that they are not breached, with the clock temporarily halted while awaiting any required responses from applicants. Although there is still room to improve the consistency of these reviews, and of course there is almost always room for administrative fine tuning, this nationalised process is currently robust and efficient. We feel that by confusing this ethics review with other aspects of research governance (described below), Petrova and Barclay significantly misrepresent the current ethics review process. For instance, the authors repeatedly use the word “ethics approvals”. Although we accept that this term is widely used with reference to research ethics committee review (and indeed the HRA uses the word “approval” to refer to both the ethics review and the governance review combined) research ethics committees themselves do not have the authority to approve projects. Instead they offer an **opinion** as to whether a piece of research is ethically acceptable if conducted in accordance with the protocol under review (see GAfREC chapter 5 [9]). It is then a subsequent governance decision whether or not to “approve” the protocol taking into account the ethics committee opinion as well as other legal and policy considerations. Petrova and Barclay perpetuate this common misunderstanding.

### Research governance

Research governance is related to research ethics, but operates under a different philosophy and using different methods. While research ethics committee examine the protocol and give an opinion in light of ethical principles, research governance focusses on the duties required of the researchers and organisations conducting the research. Such duties are often reflected in the form of laws and policies, and thus require assessment by research officers (often based in University, NHS Trust or company Research and Development (R&D) offices) with expertise in areas such as law (clinical trial regulations, radiation legislation etc.), employment contracts, insurance and indemnity, risk assessment and often local knowledge of practical issues inherent to conducting research in busy clinical environments. Here again the HRA has attempted to significantly streamline the process by introducing its nation-wide “HRA Assessment process”, something that was not yet in full swing when Petrova and Barclay were conducting their work. Indeed in response to Petrova and Barclay the HRA have stated [11]:

*We acknowledge and recognise the frustrations experienced by the researchers in the paper, which may be familiar to anyone who submitted a study before HRA Approval was implemented in 2016, but would like to ensure that readers consider this in the context of systems that were in place in 2013 and those that are in place now, over five years later.*

Having also engaged with the HRA systems as both researchers and sponsors we wish to commend the HRA for its efforts in this area of research governance, but also perhaps agree with Petrova and Barclay’s sentiments that the process is not yet operating effectively. Here the problem lies not with the HRA, which is normally able to conduct a national governance review in a timely fashion, but rather with local NHS Trust and university research departments. These departments can either be acting as sponsor (see below) or provide local approvals/permissions. As originally envisaged the HRA assessment process was designed to ensure that NHS Trust research offices only addressed matters of local capacity and capability by focusing on and resolving any issue that could be dealt with centrally, but upon the roll out of HRA Assessment we have certainly found that Hospital Trusts in particular have been guilty of creating new local processes that are responsible for the majority of delays. We therefore fully accept the data described in Table 1 of Petrova and Barclay's paper, and indeed would add to that our experience in Portsmouth (one of our researchers recorded similar data to that reported by Petrova & Barclay) where we noted over 200 email exchanges outside the HRA Assessment process when trying to initiate two projects with three different NHS trusts. It is in this local governance aspect where significant improvements have yet to be made. There is always a tension between national and local processes, but if the decision has been made to have a national level governance assessment, more can and must be done to minimise local processes.

A second element of research governance comes from interactions between the researcher and the research sponsor (whose roles are clearly defined in the *UK policy framework for health and social care research* [12]). The research sponsor role is particularly important within healthcare research as it provides the necessary institutional support to individual researchers. Interactions with the research sponsor are distinct from interactions with the HRA or other external bodies. When NHS Trusts act as sponsors they are often also the employers of researchers, and thus many of the interactions fall under normal employer-employee interactions. This is therefore a distinct role from the NHS Trust providing local project approvals. Likewise interactions with research funders, or Clinical Research Networks, either in the course of applying for research funding or subsequent contractual or policy requirements as the research is developed, registered and conducted, again represents a different part of the governance process. We feel it is unfair to include these sponsor, funding and registration processes as part of the “Research Approvals Iceberg”.

## Melting the iceberg?

Petrova and Barclay’s paper concludes with six recommendations for how the system can be improved. Many of these have currently already been actioned (as described in the HRA’s response to the article [11]), however they are right to point out problems with study classification where we agree that it can be unhelpful to distinguish “research” from “audit” and “service evaluations” [13]. However, the distinction is a matter of policy for the pragmatic reason that seeking to improve practice is an inherent part of clinical and most professional activity. Of course this does not mean that these related activities do not require close governance and ethics scrutiny, but as these activities are mostly carried out by individuals in their place of work, they are more appropriately covered under normal employment and clinical arrangements/contracts/indemnities. We do not, however, agree that there is scope for coordination between different parts of the research process such as funding applications, patient/participant involvement (PPI) reviews and ethics forms. This is because grant reviews by funding bodies are conducted well before a protocol for a clinical trial has been developed, simply because the funding is required to employ the staff to design the protocol. Likewise patient participant involvement (PPI) is focussed on both setting priorities and commenting on aspects of research design as part of protocol development. Similar to the process of engaging with the sponsor organisation, we would therefore argue that both funding and PPI interactions are part of the early research development process itself, not the “regulatory iceberg”. It is difficult to see how these types of review can be combined with the research ethics committee or governance reviews that require a finalised, well defined, protocol.

In conclusion, the paper by Petrova and Barclay provides a good opportunity to critically evaluate the research regulatory process in the UK. They are correct that the overall regulatory process can be laborious and highly frustrating, but by conflating the research ethics “opinion”, research governance “approval” aspects, and interactions with sponsors/funders/PPI groups, we feel that they have not accurately identified the parts of the system that can be most improved, and also directed unfair criticism at research ethics committees. We propose the following alternative suggestions for seeing system change:

1. Promote the distinction between research ethics and research governance
2. Encourage employers to recognise and support staff involvement in research ethics committees.
3. Ensure that the national Assessment process captures all relevant governance processes thereby minimising local processes.

## Data Availability

Not Applicable.
